# Supplementary material for: House sparrows do not show a diel rhythm in double-strand DNA damage in erythrocytes
Source: PeerJ. 2024 Oct 25;12:e18375. doi: 10.7717/peerj.18375 (PMC11514765; doi:10.7717/peerj.18375)
Supplement: Supplemental Information 7 [file peerj-12-18375-s007.docx]

Metadata for “House sparrows do not show a diel rhythm in double-strand DNA damage in erythrocytes”

Authors: Emma S. Rosen, Lily Mikolajczak, Ursula K. Beattie, L. Michael Romero

**File: “diel rhythm data.csv”** – all data for Figure 1 (Experiment 1)

| Column name | Explanation |
| --- | --- |
| Bird | Unique bird ID |
| Time | Time of sampling (written as midnight/am/pm/noon for ease of reading) |
| Time24 | Time of sampling (0-24) |
| TailMoment | DNA damage (unit is tail moment) |
| Uric acid | Uric acid reading in mg/dL |

**File: “tissue comet data.csv”** – all data for Figure 2 (Experiment 2)

| Column name | Explanation |
| --- | --- |
| Total_Light | Total amount of light within 24-hour period (either 9 or 12) |
| Type | Type of light cycle (resonance or natural) |
| Bird | Unique bird ID |
| Tissue | AF = abdominal fat  B = blood  Hi = hippocampus  Hy = hypothalamus  L = liver |
| AvgTailMoment | DNA damage (unit is tail moment) for each tissue after resonance experiment |
| Initial comets | DNA damage (in tail moments) for blood before resonance experiment |

**File: “initial v final data.csv”** – all data for Figure 3 (Experiment 2)

| Column name | Explanation |
| --- | --- |
| Light Cycle | Resonance light cycle (6:6 or 4.5:7.5) |
| Bird | Unique bird ID |
| Time | Pre or post-resonance experiment |
| Weight | Weight in grams |
| Cort | Corticosterone in ng/mL |
| Comets | DNA damage in tail moments |

**File: “testes data.csv”** – all data for Figure 4 (Experiment 2)

| Column name | Explanation |
| --- | --- |
| Total_light | Total amount of light within 24-hour period (either 9 or 12) |
| LightCycle | Resonance light cycle (6:6 or 4.5:7.5) |
| Bird | Unique bird ID |
| Weight | Testes weight in mg |
